# Myostatin and Related Factors Are Involved in Skeletal Muscle Protein Breakdown in Growing Broilers Exposed to Constant Heat Stress

**DOI:** 10.3390/ani11051467

**Published:** 2021-05-20

**Authors:** Xiumei Li, Minhong Zhang, Jinghai Feng, Ying Zhou

**Affiliations:** State Key Laboratory of Animal Nutrition, Institute of Animal Sciences, Chinese Academy of Agricultural Sciences, Beijing 100193, China; llxiumei93@163.com (X.L.); fjh6289@126.com (J.F.); 15624955881@163.com (Y.Z.)

**Keywords:** chicken, high temperature, regulatory factors, protein degradation, growth

## Abstract

**Simple Summary:**

Our results showed that constant heat stress could affect the expression of myostatin and related factors involved in skeletal muscle protein breakdown in growing broilers, resulting in a decrease in muscle protein deposition. These findings suggest a new strategy for regulating muscle protein breakdown in growing broilers, which could benefit the modern broiler industry in combating constant heat stress.

**Abstract:**

Heat stress has an adverse effect on the development of poultry farming, which has always aroused great concern. This study was carried out to investigate the protein breakdown mechanism responsible for the suppressive effect of constant heat stress on muscle growth in growing broilers. A total of 96, 29-day-old, Arbor Acres male broilers were randomly divided into two groups, a thermoneutral control (21 ± 1 °C, TC) and a heat stress (31 ± 1 °C, HS) group, with six replicates in each group and eight birds in each replicate. The trial period lasted for 14 d, and the trial was performed at 60 ± 7% relative humidity, a wind speed of <0.5 m/s and an ammonia level of <5 ppm. The results showed that the average daily feed intake and average daily gain in the HS group were distinctly lower than those in the TC group (*p* < 0.05), whereas the HS group showed a significantly increased feed conversion ratio, nitrogen excretion per weight gain and nitrogen excretion per feed intake compared to the TC group (*p* < 0.05). In addition, the HS group showed a significantly reduced breast muscle yield and nitrogen utilization in the broilers (*p* < 0.05). The HS group showed an increase in the serum corticosterone level (*p* < 0.05) and a decrease in the thyroxine levels in the broiler chickens (*p* < 0.05) compared to the TC group, whereas the HS group showed no significant changes in the serum 3,5,3′-triiodothyronine levels compared to the TC group (*p* > 0.05). Moreover, the HS group showed increased mRNA expression levels of myostatin, Smad3, forkhead box O 4, muscle atrophy F-box and muscle ring-finger 1, but reduced mRNA expression levels of the mammalian target of rapamycin, the protein kinase B and the myogenic determination factor 1 (*p* < 0.05). In conclusion, the poor growth performance of birds under constant heat stress may be due to an increased protein breakdown via an mRNA expression of myostatin and related factors.

## 1. Introduction

Heat stress (HS) is a recognized long-term problem in the poultry farming industry, and its effect on poultry production performance is a common challenge in tropical and subtropical climatic conditions and even during the summer in temperate regions [1]. It is well known that HS can decrease the production of birds [2,3,4,5]. According to the United States Department of Agriculture’s statistics, global chicken consumption reached 101.486 million tons in 2018. Poultry meat has seen a rise in its global demand because of its relatively high nutritional value, its low price and the lack of cultural or religious obstacles for its consumption [6]. Almost 50% of a broiler’s total body mass is skeletal muscle, which exhibits a strong metabolic activity as the largest protein source in the body [7]. Heat stress has a significant effect on the growth of breast muscle, which can greatly reduce the breast muscle yield [8,9,10,11,12] and result in a substantial economic loss.

The essence of muscle growth is the accumulation of protein, and the balance between the rates of protein synthesis and protein breakdown influences the muscle mass [13]. At present, studies on protein synthesis are mainly focused on the insulin-like growth factor-1 (*IGF-1*)/protein kinase B (*Akt*) pathway in mammals and birds [14,15,16]. One of the principal protein degradation systems in skeletal muscle is the ubiquitin–proteasome pathway [17]. Forkhead box O (*FoxO*) transcription factors play an important role in muscle wastage through the regulation of ubiquitin E3 ligases, muscle atrophy F-box (*MAFbx*) and muscle ring-finger 1 (*MuRF1*) [14].

Myostatin, a transforming growth factor-beta family member, is a potent negative regulator of skeletal muscle growth [18]. Many previous in vitro studies have revealed that myostatin affects mammalian muscle growth. For example, blocking the myostatin activity in mice has applications in the promotion of muscle growth [19], and myostatin can inhibit the *Akt* activation in human skeletal muscle cells [20]. In addition, McFarlane et al. (2006) reported that in cultured C2C12 muscle cells, myostatin treatment blocked the *IGF-1*/phosphatidylinositol 3-kinase/*Akt* pathway and activated *FoxO1*, leading to an increased expression of *MAFbx* and *MuRF-1*. Smad3 transcription factors are found downstream of myostatin type II receptors and can be activated by the interaction of myostatin with its receptors [21].

In birds, a recent study revealed that myostatin significantly increased the phosphorylation rate of Smad2 and the mRNA levels of *MAFbx* in a chick’s embryonic myotubes cultured at 37 °C for 2 h in vitro [22]. Several previous studies of broilers have shown that HS affected muscle growth via the expression of several genes. Acute (24 h) HS decreased the expression of the *IGF-1* and phosphatidylinositol 3-kinase R1 genes in the liver and increased the cathepsin L2 and *MAFbx* gene expression in male broilers (Cobb 500) [23]. Chronic HS decreased the muscle protein synthesis in Arbor Acres male broilers by downregulating the *IGF*-mammalian target of rapamycin (*mTOR*) signaling pathway [16]. Furukawa et al. (2016) [24] reported that short-term (0, 0.5 and 3 d) HS treatment induced a superoxide production in the muscle mitochondrial of Ross male broilers as well as a *MAFbx* gene expression and affected the signaling pathway governing the *FoxO3* activity and expression. Zuo et al. (2015) [1] reported that constant HS reduced the skeletal muscle protein deposition in broilers by decreasing the *IGF-1*, phosphatidylinositol 3-kinase and p70S6 kinase expression and increasing the *MuRF1* and *MAFbx* expression. However, whether the myostatin and related factors involved in skeletal muscle protein breakdown in growing broilers are affected by constant HS remains unclear. Therefore, the present study aimed to investigate the effects of constant HS on the growth performance, breast muscle yield, nitrogen utilization and mRNA expression of myostatin and related factors in growing broilers.

## 2. Materials and Methods

### 2.1. Birds and Treatments

One-day-old male Arbor Acres broilers were kept in one-tier cages and were maintained with administrative procedures and a standard corn–soybean-meal diet consistent with the Nutrient Requirements of Poultry (1994) for Arbor Acres broilers. At 29 d old, a total of 96 healthy Arbor Acres male chicks with similar BWs (1000 ± 70 g) were selected and randomly divided into two groups, the thermoneutral control (TC) and the HS group, which were raised in two environmentally controlled chambers. There were 6 cages (one-tier, 0.80 m × 0.80 m × 0.40 m) with 8 birds per cage in each environmentally controlled chamber (each cage served as a replicate). From the age of 29 to 42 d, the birds in the TC group were reared at a constant temperature of 21 ± 1 °C, whereas those in the HS group were reared at a constant temperature of 31 ± 1 °C. The two chambers were maintained at 60 ± 7% RH with a wind velocity of <0.5 m/s, an ammonia level of <5 ppm and a 24-h light. All broilers had ad libitum access to feed and water.

### 2.2. Sampling Collection and Chemical Analysis

Growth performance and breast muscle yield. At 42 d of age, 12 broilers from each group (two sampled birds per replicate) were randomly selected and euthanized by cervical dislocation. Their feed intake was recorded daily to calculate the average daily feed intake (ADFI). Their average daily gain (ADG) was calculated as the difference between the values of the body weight of all the birds at the beginning (29 d) and the end (42 d) of the experiment. The feed conversion ratio (FCR) was calculated as the ratio of the ADG to the ADFI. The breast muscle yield was expressed as the ratio of the breast muscle mass to the eviscerated carcass weight.

#### 2.2.1. Nitrogen Utilization and Nitrogen Excretion

The randomly selected feed from the two groups was mixed, reduced to 200 g using the quartering method and crushed. Then, the feed was placed in a sealed bag prior to being tested to detect its nitrogen intake. The excreta of all the birds in each cage was collected (each cage was a replicate) on the 14th day of the experiment (every 4 h for a total of 6 times) to detect nitrogen in the excreta. Sundries in the excreta were removed, and the excreta was weighed and then nitrogen-fixed with 10 mL of 10% H_2_SO_4_ per 100 g excreta sample. Finally, the excreta collected from one cage was mixed and 200 g of the excreta was selected using the quartering method, dried at 65–70 °C until it reached a constant weight, collected and crushed. The content of nitrogen in the feed and the excreta was determined using the Kjeldahl method [25]. The following equations were used for the calculation:

Nitrogen utilization (%) = [(nitrogen intake-nitrogen excretion)/nitrogen intake] × 100(1)

Nitrogen excretion per weight gain (%) = (nitrogen excretion/daily gain) × 100(2)

Nitrogen excretion per feed intake (%) = (nitrogen excretion/daily feed intake) × 100(3)

#### 2.2.2. Blood Measurements

Serum samples were obtained by blood centrifugation at 3000× *g* for 20 min at 4 °C, and then stored at −20 °C until the analysis took place. The serum corticosterone, 3,5,3′-triiodothyronine (T3) and thyroxine (T4) levels were measured by radioimmunoassay using a gamma radioimmunoassay counter (GC-2010, Anhui Ustczonkia Scientific Instruments Co., Ltd., Anhui, China). All procedures were conducted by following the manufacturer’s instructions.

#### 2.2.3. Regulatory Factors Gene Expression

Breast muscle samples were collected and stored at −80 °C for further analysis. Total RNA was isolated from each breast muscle sample using a TRIzol reagent (Invitrogen, Carlsbad, CA, USA) as described in the manufacturer’s instructions. A real-time quantitative PCR was carried out using a LightCycler 96 system (LightCycler 96 system, Roche, Basel, Switzerland) according to a common real-time quantitative PCR method. The mRNA levels of myostatin, Smad3, *MAFbx*, *MuRF1*, *mTOR*, *FoxO4*, myogenic determination factor 1 (*MyoD*) and *Akt* in the breast muscle were examined. The primers for the target genes were designed and confirmed based on the sequences described in GenBank, which are listed in Table 1. The *β-actin* gene was used as an internal control for normalization. The mRNA expression data were analyzed using the 2^−ΔΔCt^ method [26].

### 2.3. Statistical Analysis

The data from the present study were analyzed using a one-way ANOVA by SAS 9.2 (SAS Institute Inc., Cary, NC, USA). The ADG, ADFI, FCR, nitrogen utilization, nitrogen excretion per weight gain and nitrogen excretion per feed intake were analyzed using the cage as the experimental unit, and other indexes were analyzed by determining the mean of two sampled birds per replicate as the experimental unit (*n* = 6). The data are expressed as the means ± SD. Statistical significance was indicated at *p* < 0.05.

## 3. Results

### 3.1. Growth Performance, Breast Muscle Yield, Nitrogen Utilization and Nitrogen Excretion

**Figure 1:**
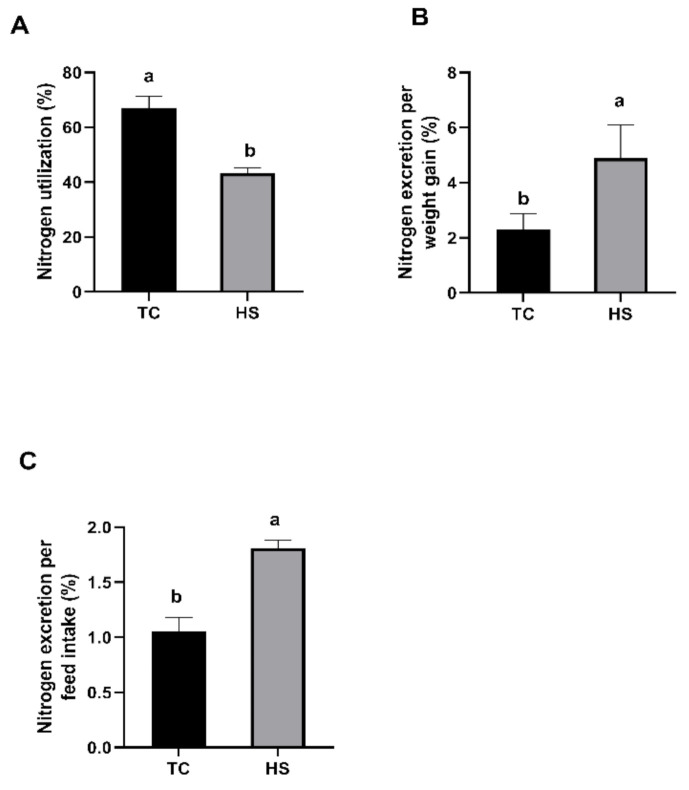
Effects of heat stress on the nitrogen utilization (**A**), nitrogen excretion per weight gain (**B**) and nitrogen excretion per feed intake (**C**) of broilers. TC, thermoneutral control group; HS, heat stress group. Each bar presents the means ± SD (*n* = 6). ^a,b^ Values with different superscripts differ significantly at *p* < 0.05.

**Figure 2:**
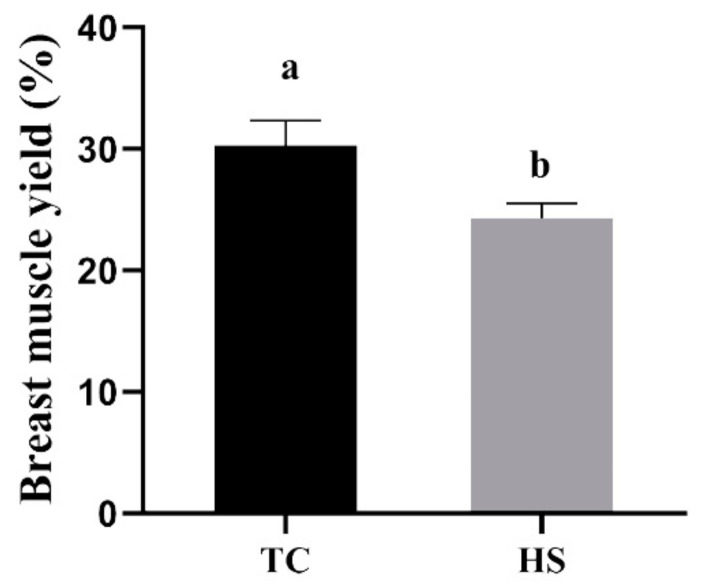
Effects of heat stress on the breast muscle yield of broilers. TC, thermoneutral control group; HS, heat stress group. Each bar presents the means ± SD (*n* = 6). ^a,b^ Values with different superscripts differ significantly at *p* < 0.05.

During the trial period, none of the birds suffered from clinical diseases, and there was no mortality. As shown in Table 2, the ADFI and ADG in the HS group were significantly lower than those in the TC group (*p* < 0.05), and the HS group had a significantly increased FCR compared to the TC group (*p* < 0.05). As shown in Figure 1A, the nitrogen utilization in the HS group was significantly lower than that in the TC group (*p* < 0.05), whereas the nitrogen excretion per weight gain (Figure 1B) and the nitrogen excretion per feed intake (Figure 1C) in the HS group were significantly higher than those in the TC group (*p* < 0.05). As shown in Figure 2, compared to those in the TC group, the broilers in the HS group showed a significantly reduced breast muscle yield (*p* < 0.05).

### 3.2. Blood Biochemical Indexes

**Figure 3:**
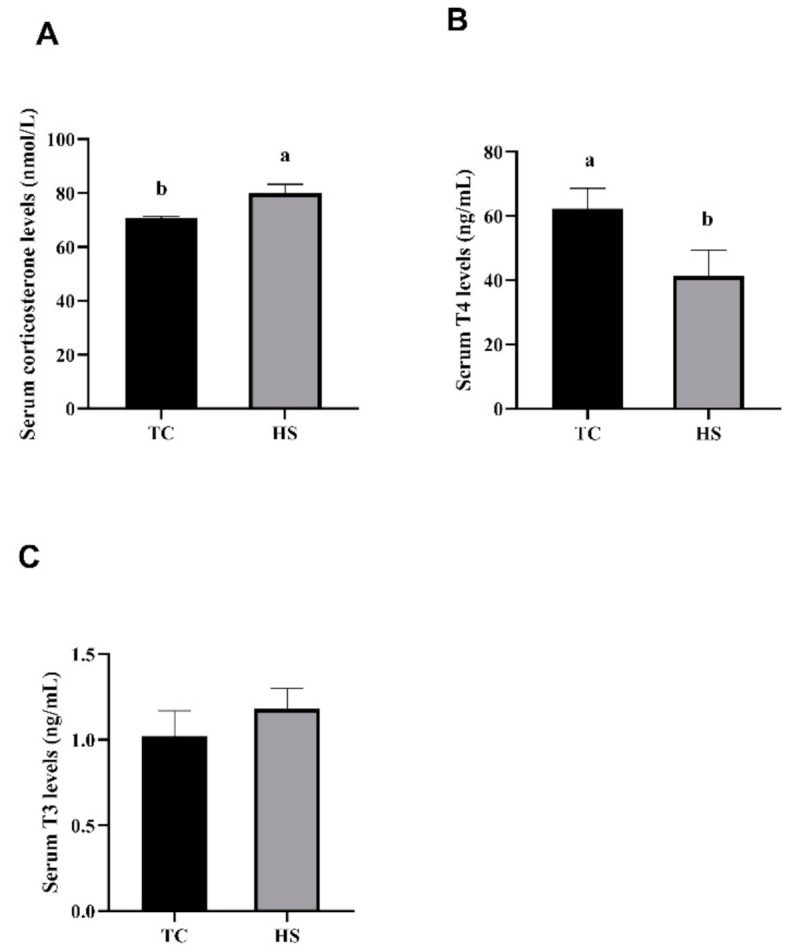
Effects of heat stress on the serum corticosterone (**A**), thyroxine (T4) (**B**) and 3,5,3′-triiodothyronine (T3) (**C**) levels in broilers. TC, thermoneutral control group; HS, heat stress group. Each bar presents the means ± SD (*n* = 6). ^a,b^ Values with different superscripts differ significantly at *p* < 0.05.

We tested the effects of HS on the blood biochemical indexes of broilers, and the results of the serum corticosterone, T4 and T3 levels in broilers are presented in Figure 3. As shown in Figure 3A, the serum corticosterone levels in the broilers in the HS group were significantly higher (*p* < 0.05) than the serum corticosterone levels in the broilers in the TC group, whereas the levels of T4 in the serum from the broiler chickens in the HS group were significantly less than the serum T4 levels in the TC group (*p* < 0.05, Figure 3B). No significant differences in the levels of T3 were observed between the TC and HS groups (*p* > 0.05, Figure 3C).

### 3.3. Regulatory Factors Gene Expression

**Figure 4:**
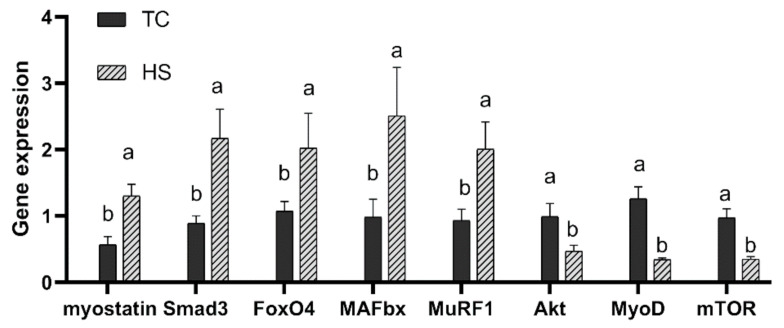
Effects of heat stress on the mRNA expression of breast muscle growth-related regulatory factors in broilers. TC, thermoneutral control group; HS, heat stress group. Each bar presents the means ± SD (*n* = 6). ^a,b^ Values with different superscripts differ significantly at *p* < 0.05. *FoxO4* = forkhead box O 4; *MAFbx* = muscle atrophy F-box; *MuRF1* = muscle ring-finger 1; *Akt* = protein kinase B; *MyoD* = myogenic determination factor 1; *mTOR* = mammalian target of rapamycin.

The gene expression of breast muscle growth-related regulatory factors in broilers exposed to constant HS was determined, and the results of the myostatin, Smad3, *FoxO4*, *MAFbx*, *MuRF1*, *Akt*, *MyoD* and *mTOR* mRNA expression levels are presented in Figure 4. As shown in Figure 4, the HS group showed significantly increased mRNA expression levels of myostatin compared to the TC group (*p* < 0.05), and the HS group showed significantly increased mRNA expression levels of Smad3, *FoxO4*, *MAFbx* and *MuRF1* as well, whereas the HS group showed significantly reduced mRNA expression levels of *Akt, MyoD* and *mTOR* (*p* < 0.05).

## 4. Discussion

Previous studies indicated that HS could affect productivity, reduce the body weight and feed intake, and increase the FCR expression of broilers [2,9,27,28]. The data in the present study revealed that the HS group showed significantly reduced ADFI and ADG values and increased FCR values compared to the TC group. These results were consistent with the findings of previous studies. HS significantly decreased nitrogen utilization and increased the nitrogen excretion per weight gain and nitrogen excretion per feed intake in this study, which revealed that HS reduced protein utilization. Kumar et al. (2017) [29] reported that a reduced nitrogen excretion is a result of an increased digestibility of protein and an increased deposition of protein in broilers. A significant increase was observed in the nitrogen excretion per weight gain and the nitrogen excretion per feed intake, showing that HS directly led to muscle protein degradation. The breast muscle is a main part of the total body, and HS directly impairs broiler production. It has been reported that HS decreased the proportion of breast muscle [10,30,31]; as expected, our results were the same as those of previous studies and indicated that HS significantly reduced the breast muscle yield of broilers. Muscle growth is the result of a rate of protein synthesis greater than the rate of protein breakdown; in heat-stressed broilers, both protein synthesis and breakdown are affected by heat exposure [32]. Based on the decreases in the ADFI, ADG, breast muscle yield and nitrogen utilization and the increases in the FCR, nitrogen excretion per weight gain and nitrogen excretion per feed intake, HS reduced protein deposition and promoted protein breakdown in the broilers. Lin et al. (2004) [33] pointed out that the reduced growth rate was related to the proteolysis that was induced by corticosterone, which could cause a reduction in animal body weight gain. T3 and T4 are the major hormones that are required to support normal growth and are known to impact almost every physiological process in chickens [34]. Studies have shown that HS reduced the plasma concentrations of T4 and T3 [35]; based on the present results, HS significantly increased serum corticosterone levels but reduced the levels of T4, as observed in previous studies [36,37,38]. HS has no effect on serum T3 levels, probably because the effect of HS on thyroid hormones is determined by many factors [39]. These endocrinological changes, which are consistent with the changes in growth performance caused by HS, could accelerate protein hydrolysis in vivo.

Many studies have reported that a high ambient temperature decreases muscle protein content [10,16,40], and Yunianto et al. (1997) [8] demonstrated that HS decreases muscle protein synthesis and accelerates protein breakdown. In the past few years, the *IGF-1*-phosphatidylinositol 3-kinase-*mTOR* signaling pathway, which is responsible for regulating the protein synthesis pathways, has been defined and studied [15,41]. Ma et al. (2018) [16] reported that chronic heat stress decreased muscle protein synthesis by downregulating the *IGF–mTOR* signaling pathway. *Akt* can phosphorylate a series of protein substrates to activate its downstream *mTOR* channels once it is activated [1]. The mammalian target of rapamycin can mediate protein synthesis through its downstream targets, and the *mTOR* pathway is known as a key signaling pathway that regulates the muscular hypertrophy process in vivo [42]. It has been reported that a member of the transforming growth factor-beta superfamily, myostatin, has a dramatically negative effect on muscle growth by binding to *Akt* in order to elicit its biological effects [18]. Forbes et al. (2006) [43] indicated that the R-Smads, Smad2 and Smad3, could be activated by myostatin to transduce signaling, which could cause the formation of complexes in the nucleus to regulate the expression of target genes through interactions with transcription coactivators or repressors. In normal chicken myotubes cultured in vitro, Smad controlled the myostatin expression [22]. The present study showed an increase in Smad3 mRNA levels in growing broilers under constant heat stress, indicating that there is a relationship between Smad3 and myostatin expression changes under constant HS, but the specific molecular changes remain to be further studied. Intracellular protein degradation occurs mainly through the ubiquitin–protein enzyme (proteasome) process, which is associated with *FoxO* transcription factors and two muscle-specific ubiquitin ligases (E3s), *MAFbx* and *MuRF1* [1,44]. The *FoxO* transcription factors play an important role in muscle atrophy. Sandri et al. (2004) [14] showed that the decreased activity of the *Akt* signaling pathway seemed to lead to an increase in the hypophosphorylated active forms of the *FoxO* transcription factors, and the *FoxO* transcription factors resulted in skeletal muscle atrophy by regulating atrophy-related genes, including *MAFbx*. In the current study, the HS group showed increased mRNA expression levels of myostatin, *FoxO4*, *MAFbx* and *MuRF1*, and reduced mRNA expression levels of *mTOR* and *Akt*. These data implied that HS may decrease broiler breast muscle growth by increasing the activity of myostatin and related factors, thus promoting muscle protein degradation. The myogenic determination factor 1 is involved in myoblast differentiation and is required for fast fiber formation [45,46]. Moreover, it has been reported that myostatin negatively regulates *MyoD* expression in muscle [47]. In the present study, we found that the mRNA expression levels of *MyoD* in breast muscle were reduced in the HS group, which indicated that HS affected breast muscle differentiation and hypertrophy, and thereby inhibited muscle growth.

## 5. Conclusions

The change in myostatin mRNA expression in growing broilers exposed to constant HS was preliminarily studied for the first time in this experiment. Therefore, we speculated that in growing broilers under high temperature conditions, protein deposition may be affected not only by the *IGF-1-Akt* signaling pathway, but also by myostatin and related factors involved in skeletal muscle protein breakdown. Moreover, further studies are required to clarify the molecular mechanism of myostatin and related factors in growing broilers under constant HS.

## Figures and Tables

**Table 1:** Primers used for quantitative RT-PCR.

Primer Name ^1^	Primer Sequence ^2^ 5′-3′	Product Size (bp)	GenBank Accession Number
*β-actin*	F: TGCTGTGTTCCCATCTATCG	150	NM_205518
	R: TTGGTGACAATACCGTGTTCA		
*mTOR*	F: AAGGATGCTGACAAACGCTATGGA	225	XM_417614
	R: ACTGACTGACTGGCTGAGTAGGAG		
myostatin	F: TACCCGCTGACAGTGGATTTC	153	NM_001001461
	R: GCCTCTGGGATTTGCTTGG		
*MyoD*	F: GGAGAGGATTTCCACAGACAACTC	113	NM_204214
	R: CTCCACTGTCACTCAGGTTTCCT		
*Akt*	F: GCTGGCATTGTTTGGCAAGATGT	215	NM_205055
	R: GCGGTTCCACTGGCTGAATAGG		
*FoxO4*	F: GCTCTTCTCACACCTGGCTCTC	186	XM_015278657
	R: TGGTTCTGCCTGCTGCTCTG		
Smad3	F: GCGTTCTGGTGCTCCATATCCTAC	192	NM_204475
	R: TCCTCTTCCGATGTGCCGTCTC		
*MAFbx*	F: CAGTGAGCCAGCCTCTTGTGATG	114	NM_001030956
	R: TTCAGCCAGTGTGACAGTCTCAGT		
*MuRF1*	F: GCGAGCAGGAGGACAAGACAAG	240	XM_424369
	R: CAAGACTGACTGTGAAGGCATCCA		

^1^*β-actin*, beta-actin; *mTOR*, mammalian target of rapamycin; *MyoD*, myogenic determination factor 1; *Akt*, protein kinase B; *FoxO4*, forkhead box O 4; *MAFbx*, muscle atrophy F-box; *MuRF1*, muscle ring-finger 1. ^2^ F, forward; R, reverse.

**Table 2:** Effects of heat stress on the growth performance of broilers ^1^.

Items	TC ^2^	HS ^2^	*p*-Value
ADFI (g)	153.30 ^a^ ± 1.17	127.30 ^b^ ± 1.76	0.02
ADG (g)	82.08 ^a^ ± 0.53	62.07 ^b^ ± 1.01	<0.01
FCR	1.87 ^b^ ± 0.01	2.05 ^a^ ± 0.01	<0.01

ADFI = average daily feed intake; ADG = average daily gain; FCR = ADFI/ADG. ^1^ All the means are reported as the means ± SD (*n* = 6). ^2^ TC, thermoneutral control group; HS, heat stress group. ^a,b^ Values with different superscripts differ significantly at *p* < 0.05.

## Data Availability

Not Applicable.
